# Machine Learning–Based Detection of Prophylactic Indication Mentions for Stress-Related Mucosal Disease in Chinese Clinical Progress Notes: Text Classification Study

**DOI:** 10.2196/103192

**Published:** 2026-11-02

**Authors:** Wei Jiang, Shuo Wang, Haonan Liang, Luyao Xu, Jie Hu

**Affiliations:** 1 Department of Pharmacy, Quzhou People's Hospital, The Quzhou Affiliated Hospital, Wenzhou Medical University, 100 Minjiang Avenue, Quzhou, Zhejiang, China, 86 0570 3121128; 2 Department of Pharmacy, Yidu Central Hospital of Weifang, Weifang, Shandong, China; 3 Department of Pharmacy, Rongcheng City People's Hospital, Rongcheng, Shandong, China

**Keywords:** natural language processing, NLP, text classifier, stress-related mucosal disease, SRMD, algorithm, prophylactic indication detection

## Abstract

**Background:**

Machine learning has been extensively applied in medical prediction; however, existing machine-assisted pharmacotherapy review systems predominantly rely on structured, codifiable data while neglecting unstructured natural language clinical narratives, thereby allowing latent medication risks to remain undetected.

**Objective:**

To address the limitations of structured data–based review, this study proposes and validates a machine learning framework grounded in text classification for the automated identification of “prophylactic” vs “nonprophylactic” medication indications within clinical narratives. As a result, it aims to assist in the automated detection of prophylactic indication mentions for stress-related mucosal disease in clinical narratives as a preliminary step toward comprehensive medication-use review.

**Methods:**

A total of 3695 progress notes were independently annotated by 2 clinical pharmacists to construct a binary dataset for classifying prophylactic medication indication for stress-related mucosal disease, yielding an interannotator agreement κ of 0.8934. Comparative experiments were conducted using traditional machine learning models, including logistic regression, support vector machine, random forest, CatBoost, and LightGBM, alongside TextCNN and a Chinese pretrained BERT model. The dataset was split into training, validation, and test sets on a patient-wise basis, and model evaluation was conducted on the independent test set. SHAP (Shapley additive explanations) analysis was used to achieve model interpretability and elucidate decision-making processes, ultimately enabling automated indication detection of prophylactic proton pump inhibitor administration.

**Results:**

All models and strategies demonstrated efficacy in medical text classification tasks. In a single-center cohort of 66 patients (3695 notes, 11.0% positive), the CatBoost model with balanced class weights achieved a note-level *F*_*1*_-score of 0.727 (precision=0.693; recall=0.765) and a patient-level *F*_1_-score of 0.889 (precision=0.800; recall=1.000) on the independent test set. At the note level, CatBoost achieved a higher *F*_1_-score than TextCNN (*F*_1_-score=0.718) and BERT (*F*_1_-score=0.628).

**Conclusions:**

In summary, the text classifier constructed through machine learning and natural language processing effectively extracts prophylactic medication indications for stress-related mucosal disease from unstructured progress notes. Combined with patient-level data partitioning, standardized annotation protocols, and an ethics-compliant framework, this approach provides an interpretable preliminary screening tool for identifying potential prophylactic indications to support subsequent pharmacist adjudication.

## Introduction

### Background

Stress-related mucosal disease (SRMD) is a superficial lesion most commonly observed in the acid-producing regions of the gastric mucosa; in the intensive care unit, its predisposing factors include prolonged mechanical ventilation, coagulation disorders, shock, severe trauma, surgery, burns, and multiple organ failure [1]. Proton pump inhibitors (PPIs) are frequently used for both prophylaxis and treatment of SRMD; however, these agents are often prescribed beyond approved indications or for conditions with limited therapeutic benefit, rendering the issue of PPI overuse a matter of considerable clinical concern [2-4].

Prescribing errors and adverse drug events are associated with substantial morbidity and mortality and contribute substantially to avoidable health care resource waste and unnecessary medical expenditure. It is widely recognized that although individual operational errors constitute the immediate precipitating factors for prescribing errors and adverse drug events, their fundamental etiology lies in systemic failures of computerized health information systems [5,6]. In 2010, the Ministry of Health of the People’s Republic of China promulgated the “Hospital Prescription Review Management Standards (Trial),” [7] which mandated that hospitals arrange trained pharmacists to conduct random sampling reviews of at least 100 prescriptions monthly. Nevertheless, current review practices are constrained by limited scope of inspection and delayed regulatory oversight [8,9].

Electronic health records (EHRs) constitute comprehensive archival systems that document patients’ health status across multiple dimensions, encompassing medical history, therapeutic interventions, and diagnostic outcomes. EHR coding typically comprises 3 distinct datasets: diagnostic, procedural, and medication domains [10]. In addition to coded structured data, EHRs commonly retain electronic medical record (EMR) information rendered in natural language, such as progress notes. Certain medication indications are frequently embedded within these unstructured natural language texts, the extraction of which typically necessitates manual mining by clinical pharmacists. Although several hospitals have implemented knowledge-based approaches for prescription appropriateness screening, the review data sources for such methods are predominantly derived from structured coded data within EHRs, with insufficient use of critical features contained in natural language EMR narratives [11-13].

Machine learning systems autonomously formulate rules during the training process based on empirical data, thereby achieving generalization from previously learned datasets. Empirical evidence has demonstrated that the integration of health care and machine learning enhances patient care quality, improves operational efficiency within the medical industry, and reduces costs, ultimately facilitating the development of more intelligent, rapid, and efficient health care systems [14]. Existing reviews have indicated that the integration of AI tools such as machine learning into hospital clinical pharmacy practice holds substantial potential for application value and developmental prospects [15].

### Aim of This Study

This study aims to use machine learning methodologies through a binary text classification approach to identify potential SRMD characteristics within EMRs, determining whether prophylactic indications for SRMD are present in clinical narratives, thereby assisting pharmacists in detecting mentions of these potential indications within EMR progress notes. It should be emphasized that this study constitutes indication mention detection rather than comprehensive rational-use review; the latter would additionally require linkage to actual medication orders, dosing, and pharmacist adjudication.

## Methods

### Study Design, Data Source, and Annotation

EMRs of patients receiving PPIs were collected and analyzed segment by segment, yielding a total of 3695 records. A manual annotation approach was used, with records meeting prophylactic medication criteria for SRMD designated as 1 and those without such characteristics designated as 0. The inclusion criteria explicitly required the presence of any of the following fourteen items in the text: (1) mechanical ventilation exceeding 48 hours or extracorporeal life support, (2) coagulation disorders or antiplatelet therapy, (3) history of gastrointestinal ulceration or hemorrhage, (4) severe craniocerebral or cervical spinal cord trauma, (5) severe burns, (6) severe trauma or multiple injuries, (7) surgical procedures exceeding 3 hours, (8) acute renal failure or renal replacement therapy, (9) chronic hepatic disease or acute hepatic failure, (10) acute respiratory distress syndrome, (11) shock or sustained hypotension, (12) sepsis, (13) cerebrovascular or cardiovascular accidents, or (14) severe psychological stress. These criteria were derived from the “Guiding Principles for Clinical Application of Proton Pump Inhibitors (2020 Edition)” issued by the National Health Commission of the People’s Republic of China [16].

### Ethical Considerations

This study was reviewed and approved by the Medical Ethics Review Committee of Quzhou People's Hospital; The Quzhou Affiliated Hospital, Wenzhou Medical University (approval number: 2026‐010; approval date: January 7, 2026) and was registered on the Chinese Medical Research Registration and Filing Information Platform (filing number: MR-33-26-002512; registration number: ChiCTR2600126317).

This was a retrospective secondary analysis of existing EMR data. The original records were generated as part of routine clinical care rather than for research purposes. The requirement for informed consent was waived by the ethics committee because the study involved no contact with participants and used only deidentified data; the ethics committee approved the secondary analysis without additional informed consent. No compensation was provided, as this was a retrospective analysis of routinely collected clinical data with no participant contact.

All records were deidentified before annotation and analysis: directly identifying information (eg, names, medical record numbers, and identification numbers) was removed, and each patient was assigned a coded identifier. The datasets used for modeling therefore contained no information capable of identifying individual patients, and access to the source data was restricted to authorized study personnel. All procedures were performed in accordance with the Declaration of Helsinki and relevant institutional guidelines.

No images or materials in this manuscript or its supplementary files permit the identification of individual participants; all figures are aggregate statistical graphics, and the clinical note excerpts quoted in the error analysis were deidentified and contain no information from which any individual could be identified. This manuscript does not contain any individual person's data, images, or videos that would require consent for publication.

### Dataset Partitioning and Term Frequency–Inverse Document Frequency Vectorization

The collected records were randomly partitioned into training, validation, and test sets at a ratio of 7:1.5:1.5, with all records from individual patients allocated exclusively to a single data subset. After splitting, the 3 subsets were saved as separate files. To prevent data leakage, term frequency–inverse document frequency (TF-IDF) vectorization was performed in a strictly leakage-free manner: the vectorizer was fitted exclusively on the training set using the specified parameters (n-gram range 1‐2, min_df=2, max_df=0.9, sublinear_tf=True). The fitted vectorizer was then used to transform the validation and test sets (without refitting), ensuring that no test-set frequency information influenced the feature representation and that all subsets shared an identical vocabulary space.

### Model Training, Threshold Selection, and Cross-Validation

During the model training phase, only the training and validation set files were loaded. The optimal classification threshold was determined by selecting the decision threshold that maximized the positive class *F*_1_-score on the validation set, and this threshold was fixed in a configuration file for use during the testing phase. The test set file was read only once by an independent inference script in the final evaluation stage, ensuring that the test data did not participate in model construction or optimization in any form.

Because the independent test set was reserved for final evaluation, repeated stratified group 5-fold cross-validation (5 independent repeats, 25 total iterations) was performed on the 56 patients in the training and validation sets. In each outer iteration, the training fold was further partitioned into inner-training and inner-validation portions on a patient-wise basis (stratified by the presence of positive notes). Within each outer training fold, the class-weighting strategy (balanced or 1:5) and key hyperparameters (learning rate, tree depth, and L2 leaf regularization) were reselected by deterministic grid search (24 candidate configurations; up to 1500 iterations with early stopping on the inner-validation *F*_1_-score), and the decision threshold was selected by grid search (step=0.01) on the inner-validation portion only. The TF-IDF vectorizer was refitted on the inner-training notes of each fold. Thus, no information from the outer test folds influenced hyperparameter, class-weight, or threshold selection. To quantify uncertainty at the patient level, a cluster bootstrap was applied to the pooled out-of-fold predictions: within each repeat, patients (together with all of their notes) were resampled with replacement 1000 times, and the 95% CIs were averaged across the 5 repeats. The 25 fold-level estimates are reported as descriptive means and SDs.

### Comparative Experiments and Evaluation Metrics

The original natural language text records were segmented into words using the third-party Chinese word segmentation Jieba library [17]. The Chinese stop-word list was obtained from the open-source repository *cn_stopwords.txt* on GitHub [18]. Comparative experiments were subsequently performed using traditional machine learning models (random forest, logistic regression, support vector machine, LightGBM, and CatBoost) alongside deep learning baseline models (TextCNN and the Chinese pretrained model chinese-bert-wwm-ext).

A comparative strategy between traditional machine learning models and deep learning baseline models was established. The machine learning algorithms were combined with TF-IDF to obtain vector representations of the segmented text [19]. The TF-IDF vectorization parameters were configured as follows: n-gram range set to (1,2) to capture both unigrams and bigrams; min_df=2 to exclude terms appearing in fewer than 2 documents; max_df=0.9 to eliminate high-frequency terms present in more than 90% of documents; and sublinear_tf=True to apply logarithmic smoothing to term frequencies. For the traditional machine learning models, class weights and key hyperparameters were tuned with the objective of maximizing the positive-class *F*_1_-score on the validation set. The predictive performance of the CatBoost, TextCNN, and BERT models when processing raw text was compared against the TF-IDF–based machine learning models. TextCNN adopted a multiscale 1D convolutional architecture, while BERT used hfl/chinese-bert-wwm-ext (12 layers, 768 dimensions). Both models were implemented in PyTorch, and the training objective was to maximize the positive-class *F*_1_-score on the validation set. Hyperparameters were automatically determined using Optuna with a tree-structured Parzen estimator sampler. The final hyperparameters for TextCNN were as follows: emb_dim=64, num_filters=200, dropout=0.2, lr=0.001, batch_size=16, max_len=512, and filter_sizes=[2,3,5]. For BERT, the hyperparameters were the following: max_len=128, batch_size=8, lr=1.2551e-05, dropout=0.5, weight_decay=0.06, warmup_ratio=0.15, and epochs=5.

Meanwhile, within the vector-based modeling framework, the effect of conventional class weight settings was further compared to examine the interference of positive-negative class imbalance on model performance. By comparing the positive-class *F*_1_-score, precision-recall area under the curve (PR-AUC), receiver operating characteristic area under the curve (ROC-AUC), and generalization gap of each model on the test set, the modeling effectiveness of different algorithms and text processing strategies for medical text classification tasks was systematically evaluated.

In addition to note-level metrics, patient-level performance was evaluated to account for the nonindependence of multiple notes contributed by the same patient. A patient was classified as positive if the predicted probability of any of their notes exceeded the threshold, which was determined on the validation set, and the patient-level ground truth was defined as the presence of at least 1 positively annotated note within that patient’s records. Patient-level precision, recall, and *F*_1_-score were then computed based on these aggregated patient-level predictions, reflecting the clinically relevant unit of analysis for pharmacist screening workflows.

Model calibration was assessed using the Brier score, with lower values indicating better calibration and predictive performance. A well-calibrated model should have a Brier score close to 0. Decision-curve analysis was performed to evaluate clinical utility across probability thresholds, comparing the net benefit of using the model against the strategies of reviewing all notes (treat-all) or reviewing none (treat-none).

As a baseline reflecting conventional rule-based screening practice, a rule-based classifier was constructed by translating the 14 guideline inclusion criteria into keyword-matching rules, including regular-expression parsing of operative time stamps for the greater-than-3-hour surgery criterion. Because a rule-based system can only encode terminology observed in previously encountered documents and cannot anticipate future documentation variability, the keyword dictionary was derived from the guideline criteria and the documentation patterns observed in the training corpus. The baseline labeled a note as positive if any rule was triggered and was evaluated on the same independent test set with identical patient-level aggregation.

### Shapley Additive Explanations–Based Model Interpretability and Error Analysis

Model interpretability was assessed using SHAP (Shapley additive explanations) [20]. For the best-performing traditional model (CatBoost with balanced class weights), TreeSHAP (tree-based Shapley additive explanations) values were computed for the independent test set to quantify each feature’s contribution to the log-odds of the positive class. Features were ranked by mean absolute SHAP value, and a beeswarm plot was generated to illustrate the direction and magnitude of local effects. For the international readership, the top-ranked Chinese terms were translated into English, and the original Chinese terms are reported alongside their translations in Table 1.

**Table 1.** Mapping of top 10 SHAP (Shapley additive explanations) features to guideline-defined stress-related mucosal disease indications. Features are presented in Chinese with their English translations in parentheses.

Feature	Guideline match	Guideline item
骨折 (fracture)	Indirect	Item 6
出血 (bleeding)	Indirect	Item 3
损伤 (injury)	Indirect	Item 6
抗凝 (anticoagulation)	Direct	Item 2
脑梗死 (cerebral infarction)	Indirect	Item 13
感染 (infection)	Indirect	Item 12
外伤 (trauma)	Direct	Item 6
抗感染 (anti-infection)	Indirect	Item 12
半月板 损伤 (meniscus injury)	Indirect/artifact	Item 6
术后 (postoperative)	Indirect	Item 7

To determine whether the classifier captured clinically meaningful context or merely relied on surface-level keyword matching, all false-positive and false-negative notes on the independent test set (as determined by the fixed decision threshold) were independently reviewed by 2 clinical pharmacists. Representative cases were selected to illustrate typical failure modes, and the note excerpts are reported in the original Chinese together with English translations.

## Results

### Dataset Characteristics

The baseline characteristics of the study cohort are summarized in Table 2. The patient-wise split resulted in a balanced distribution of positive notes across subsets (Table 3). A total of 3695 paragraphs from inpatient progress notes were enrolled in this study, comprising 407 (11.0%) paragraphs with prophylactic medication indications for SRMD and 3288 (89.0%) paragraphs without such indications. All data were derived from hospital inpatient EMRs. Two clinical pharmacists qualified for prescription review independently annotated all texts, achieving a κ of 0.8934; records with annotation discrepancies were resolved through consensus discussion to establish final agreement.

**Table 2.** Dataset characteristics at note level and patient level.

Characteristic	Value
Patient-level characteristics (n=66)	
Age (years), mean (SD)	62.9 (18.0)
Sex, n (%)	
Male	35 (53)
Female	31 (47)
Ward type: general ward, n (%)	66 (100)
Patients with ≥1 positive note, n (%)	53 (80)
Patients with all negative notes, n (%)	13 (20)
Total notes per patient, mean (SD)	56.0 (32.0)
Total text length per patient (characters), mean (SD)	5591.2 (4624.3)
Note-level characteristics (n=3695)	
Notes with SRMD^a^ prophylaxis indication, n (%)	407 (11)
Notes without indication, n (%)	3288 (89)
Average length per note (characters), mean (SD)	99.9 (211.1)^b^

a SRMD: stress-related mucosal disease.

b The note-level SD reflects high between-patient variability in note length, as notes within the same patient are nonindependent.

**Table 3.** Partitioning of patients and notes across training, validation, and test sets.

Characteristic	Training set	Validation set	Test set	Total
Patients, n	46	10	10	66
Notes, n	2558	566	571	3695
Positive notes, n (%)	239 (9.3)	100 (17.7)	68 (11.9)	407 (11.0)
Negative notes, n (%)	2319 (90.7)	466 (82.3)	503 (88.1)	3288 (89.0)
Patients with ≥1 positive note, n (%)	36 (78)	9 (90)	8 (80)	53 (80)

### Model Performance on the Independent Test Set

To comprehensively compare the classification performance, generalization ability, and adaptability to class-imbalance sampling strategies of different models on the dataset used in this study, a visual analysis was conducted from the following perspectives: ranking of positive-class *F*_1_-score on the test set, generalization error, performance distribution, precision-recall trade-off, and the correspondence between validation and test performance.

The test set positive-class *F*_1_-score rankings and generalization errors for each model and strategy combination are presented in Figure 1A and 1B, respectively. The CatBoost model based on TF-IDF vectorization achieved an *F*_1_-score of 0.727 under the balance strategy, with precision of 0.693, recall of 0.765, average precision (AP) of 0.751, and area under the curve (AUC) of 0.935, outperforming all other configurations. The TextCNN model achieved the second-highest *F*_1_-score of 0.718 (AP=0.797; AUC=0.956), while the pretrained BERT model achieved a test *F*_1_-score of 0.628 (AP=0.715; AUC=0.934) with the largest generalization gap of 0.246. Traditional linear models and random forest consistently underperformed gradient boosting ensemble models. Furthermore, the generalization gaps varied considerably across models, ranging from 0.151 (CatBoost balanced) to 0.246 (BERT), with certain configurations of RandomForest, LogisticRegression, and BERT exhibiting gap values exceeding 0.23, indicating substantial performance degradation on unseen patients.

**Figure 1.**
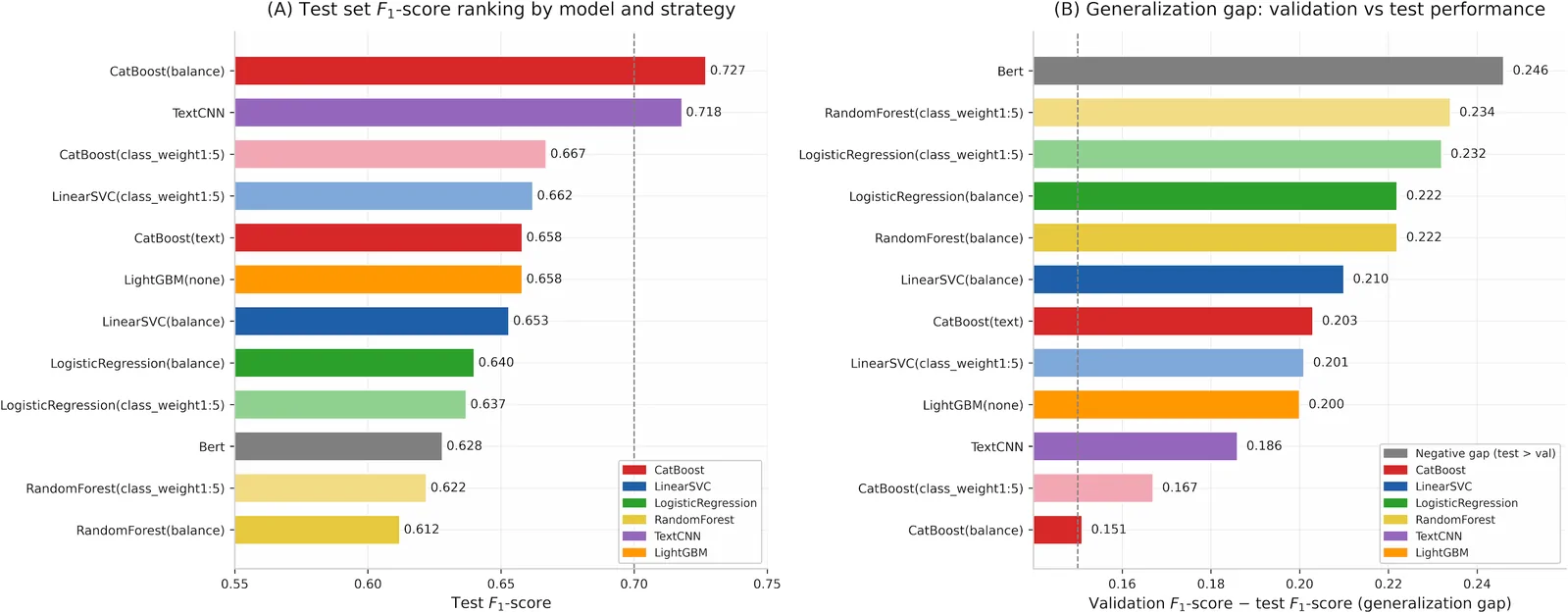
Comprehensive comparison of (A) model performance and (B) generalization capability for each model and strategy combination.

To address the nonindependence of notes within patients, patient-level performance was additionally evaluated. A patient was classified as positive if the predicted probability of any of their notes exceeded the threshold (0.46). At the patient level, the CatBoost model achieved an *F*_1_-score of 0.889, precision of 0.800, and recall of 1.000 on the 10-patient test set (Table 4).

**Table 4.** Performance metrics of the best-performing CatBoost model (balanced strategy) on the independent test set.

Metric	Note-level	Patient-level
Precision	0.693	0.800
Recall	0.765	1.000
*F*_1_-score	0.727	0.889
Specificity	—^a^	0.0^b^

a Not applicable.

b Only 2 negative patients were present in the test set; this specificity estimate is extremely imprecise and should be interpreted with caution.

At the patient level, the model identified all 8 positive patients but also flagged both negative patients (true negative=0; false positive=2), corresponding to a patient-level specificity of 0.0% (0/2). Because only 2 patients were negative in the test set, the patient-level specificity estimate is extremely imprecise and should be interpreted with caution. We therefore additionally report cross-validated patient-level performance (precision=0.864, 95% CI 0.766‐0.953) as a complementary reference.

As a reference for conventional practice, the rule-based baseline achieved a note-level precision of 0.571, recall of 0.471, and *F*_1_-score of 0.516, missing 36 (53%) of 68 positive notes while generating a false-alert count comparable to that of the CatBoost model (24 vs 23), indicating that rule-based screening would leave more than half of the indicated notes undetected at a similar reviewing workload. At the patient level, the rule-based baseline produced a true positive of 7, false positive of 0, false negative of 1, and true negative of 2 (precision=1.000; recall=0.875; *F*_1_-score=0.933), whereas the CatBoost model produced a true positive of 8, false positive of 2, false negative of 0, and true negative of 0 (precision=0.800; recall=1.000; *F*_1_-score=0.889). Given that only 2 patients were negative in the test set, patient-level estimates are unstable; in a note-level screening workflow, where the note is the unit of review and missing a positive note carries a higher cost than reviewing an additional negative note, the note-level comparison is more operationally meaningful.

Complete performance metrics for all model configurations, including precision, recall, *F*_1_-score, ROC-AUC, PR-AUC, and confusion-matrix values, are provided in Multimedia Appendix 1.

### Cross-Validation and Calibration

Under this fully nested procedure, the CatBoost model achieved a mean note-level *F*_1_-score of 0.717 (SD 0.059; patient-level bootstrap 95% CI 0.659‐0.774), precision of 0.707 (95% CI 0.638‐0.773), recall of 0.734 (95% CI 0.667‐0.798), ROC-AUC of 0.953 (SD 0.017), and PR-AUC of 0.756. At the patient level, the mean *F*_1_-score was 0.890 (SD 0.062; 95% CI 0.821‐0.954), with a precision of 0.864 (95% CI 0.766‐0.953) and recall of 0.925 (95% CI 0.843‐0.991). These estimates were consistent with the independent test-set results. The patient-level cluster bootstrap yielded appropriately wider intervals than fold-level resampling, reflecting the nonindependence of notes within patients. The selected configurations varied across folds (balanced vs 1:5 class weighting; tree depth 4 vs 6), indicating that per-fold reselection captured genuine instability in hyperparameter choice under the limited sample size rather than a fixed configuration.

Figure 2A illustrates the precision-recall trade-off for each model and strategy combination on the test set. The performance distributions varied substantially across different models and strategies. The CatBoost model clustered predominantly in the high *F*_1_-score region across most configurations, demonstrating favorable comprehensive discriminative capability. Although the RandomForest model achieved the highest recall under specific configurations, its precision was markedly reduced, indicating pronounced directional bias in positive-negative sample identification. Figure 2B presents the comparative distribution of validation set vs test set *F*_1_-scores for each model. Overall, the majority of models exhibited superior performance on the validation set compared to the test set, manifesting varying degrees of generalization performance degradation.

**Figure 2.**
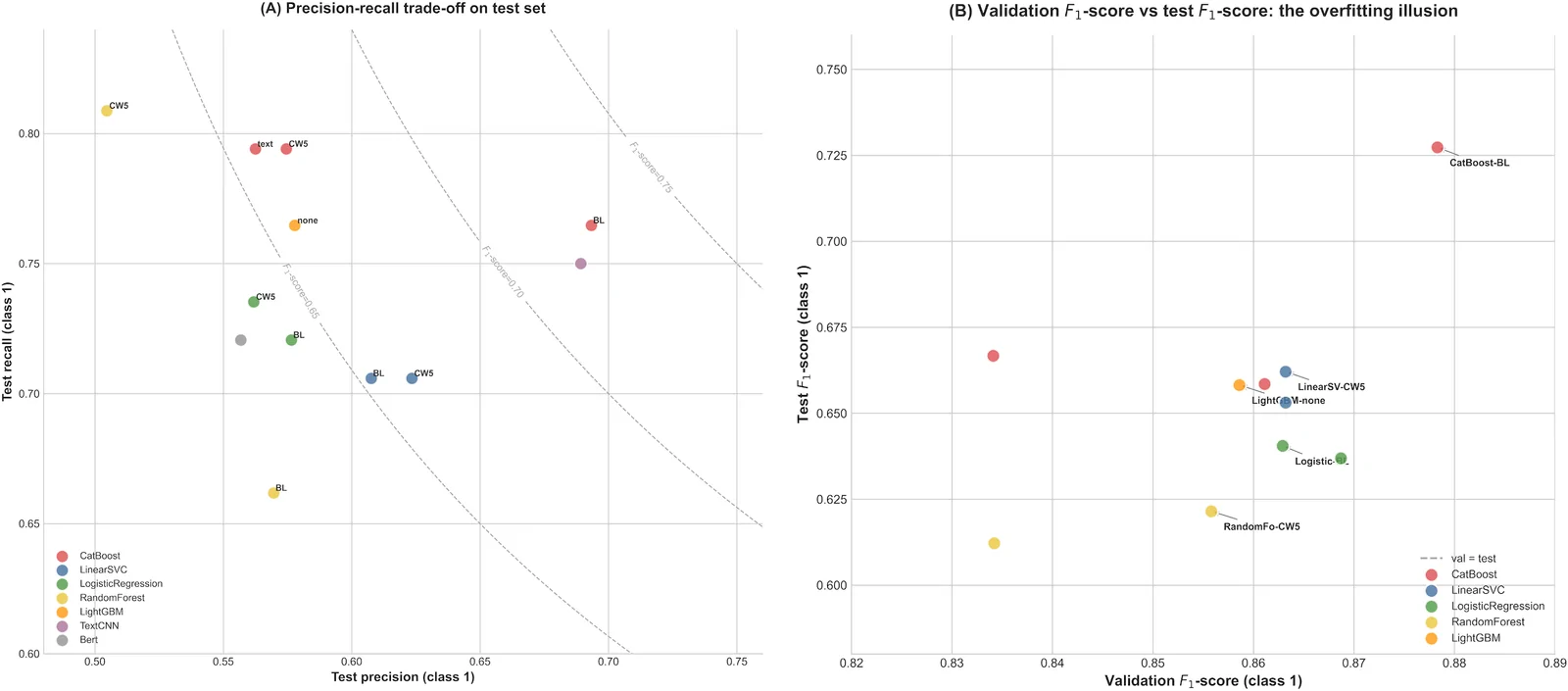
(A) Precision-recall trade-off of test set and (B) overfitting analysis comparing validation set and test set *F*_1_-scores for each model. The data labels represent strategy abbreviations across different algorithms, with the following specific designations: abbreviations prefixed with “CW” indicate the use of class weighting, whereas “CW5” denotes a weight ratio of 1:5; “BL” indicates balanced weight configuration.

The CatBoost model exhibited moderate calibration, with a Brier score of 0.052; residual miscalibration is likely attributable to the limited test-set sample size. Decision-curve analysis showed that the model achieved positive net benefit across clinically relevant probability thresholds, with net benefits of 0.084 and 0.075 at thresholds of 0.2 and 0.3, respectively, exceeding both the treat-all strategy (net benefit of −0.101 and −0.258, respectively, at the same thresholds) and the treat-none strategy (net benefit=0). Given the small test set and low event rate (68/571, 11.9% positive notes), these estimates should be interpreted as preliminary, and prospective evaluation in larger cohorts with explicit cost-benefit framing is needed to confirm clinical utility.

### SHAP Feature Analysis and Error Analysis

To facilitate comprehension for an international readership, the Chinese feature terms generated after Jieba segmentation were translated into English, whereas the original model inputs remained entirely in Chinese. Figure 3 presents the interpretability analysis results based on SHAP for the optimal traditional machine learning model, namely CatBoost with balanced class weighting strategy. The left panel displays the ranking of the top 10 features by mean absolute SHAP values, quantifying the global contribution of each feature to model predictions. The right panel presents a beeswarm plot illustrating the distribution characteristics of SHAP values across individual samples, where the color gradient denotes feature value magnitude, and the horizontal position of each data point reflects the direction and magnitude of local influence exerted by that feature on model output. SHAP values are expressed on the log-odds scale.

**Figure 3.**
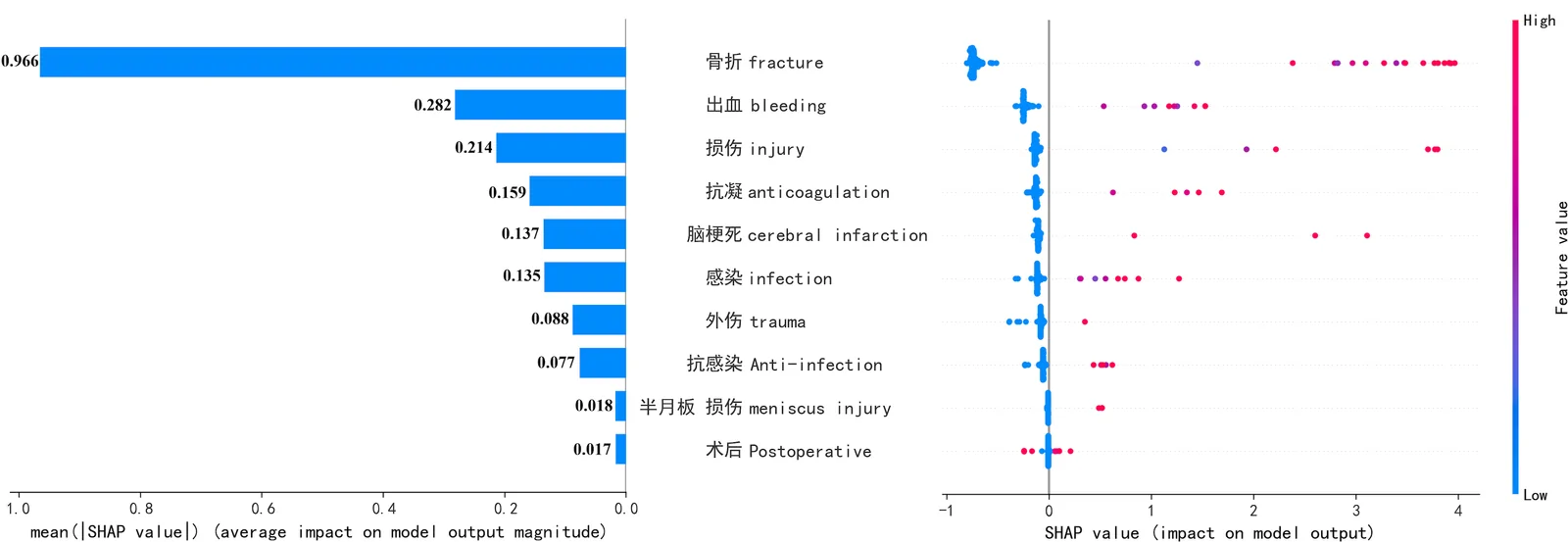
Key feature analysis based on SHAP (Shapley additive explanations).

To assess whether the model learned clinically meaningful context or merely relied on keyword matching, representative false positive and false negative cases from the test set were manually reviewed. For a false positive case, a note describing an active intracranial bleeding event stated, “左侧裂池区及左侧部分脑沟内可疑密度增高，蛛网膜下腔少量出血可疑” (suspected subarachnoid hemorrhage in the left Sylvian fissure). The model assigned a high positive probability (0.990), likely triggered by the term “出血” (bleeding). However, this text represented an acute computed tomography finding rather than a history of gastrointestinal ulceration or hemorrhage (guideline item 3) or any other SRMD prophylactic indication. This suggests that the model associated surface-level hemorrhage-related tokens with positive labels without distinguishing between active bleeding events and historical prophylactic risk factors.

As an example of a false negative case, a perioperative note documented, “手术开始时间：2024年01月02日 13时00分 手术结束时间：2024年01月02日 18时00分” (surgery start time: 1 PM; end time: 6 PM). The procedure duration was 5 hours, satisfying the greater-than-3-hour surgical threshold (guideline item 7). However, the model assigned a low probability (0.040), revealing that the TF-IDF–based classifier lacked temporal reasoning capability and could not parse procedural time stamps to calculate operative duration. These cases collectively indicate that the model relies on surface-level keyword correlation for some predictions, while lacking structured reasoning for implicit or numerically encoded indications.

## Discussion

### Principal Findings

The main finding of this study was that, among 7 compared models, CatBoost with balanced class weights achieved the highest note-level *F*_1_-score (0.727) for detecting prophylactic SRMD indication mentions in a highly imbalanced Chinese clinical note dataset, outperforming both deep learning baselines (TextCNN and BERT) in generalization stability.

Direct quantitative comparison with existing studies is inherently limited due to variations in task definition, dataset characteristics, and class distribution. Zhang et al [21] used BERT combined with TextRNN for multilabel classification of cancer literature, reporting an *F*_1_-score as high as 0.903. However, their task was based on structured English abstract texts with relatively balanced label distribution. The present study focused on a highly imbalanced binary classification task of Chinese clinical progress notes: at the record level, only 11.0% of paragraphs were annotated with prophylactic medication indications for SRMD, indicating severe record-level imbalance. Under this challenging scenario, the pre-trained BERT model achieved an AP of 0.715 and an AUC of 0.934. The TextCNN model achieved a higher AP of 0.797 and AUC of 0.956, though its generalization gap (0.186) was slightly larger than that of CatBoost. More importantly, the optimal model in this study, the CatBoost model optimized with balanced class weighting strategy, obtained AP=0.751, an *F*_1_-score of 0.727, precision=0.693, recall=0.765, and AUC=0.935, demonstrating the best balance between discriminative performance and generalization stability (gap=0.151). These results demonstrate that systematically tuned traditional machine learning models can effectively address clinical text classification problems under severely imbalanced conditions, providing a lightweight and interpretable alternative for clinical scenarios with limited computational resources, rapid future integration requirements, or interpretability demands. This study furnishes a systematic algorithmic comparison benchmark for prophylactic indication detection from Chinese EMR texts, offering a reproducible performance reference for subsequent investigations.

The comparison with the rule-based baseline illustrates a fundamental limitation of knowledge-engineered screening systems: rules can only encode terminology that has already been observed, whereas natural language documentation varies continually across clinicians, departments, and time. In this study, even though the keyword dictionary was induced from the guideline criteria and the observed training corpus, the baseline missed 36 of 68 (53%) positive notes, including severe trauma described only in physical-examination findings (eg, a deep neck laceration with exposed vertebrae) rather than with the anticipated terms such as “trauma” or “injury.” The learned model, by contrast, captures statistical regularities from the entire training corpus and generalizes to unanticipated phrasings. Notably, the baseline did capture 1 (1.5%) positive note that the model missed—the greater-than-3-hour surgery encoded as time stamps—which suggests that the 2 approaches are complementary: rules guarantee literal compliance with explicit criteria, while statistical models provide coverage of documentation variability. Maintaining a rule-based system would require continuous manual dictionary updates as documentation practices evolve, whereas the presented framework requires only retraining on newly annotated data.

It is noteworthy that health care scenarios impose exceptionally high demands on decision transparency. Prescriptions flagged by the model must provide comprehensible and verifiable rationales to pharmacists or physicians; if the system merely outputs risk scores without specific indication, clinical personnel will be unable to assess the accuracy of such flags, thereby diminishing system adoption rates [22]. Therefore, model interpretability constitutes not merely a technical optimization but an essential prerequisite for ensuring medical safety and regulatory compliance. Researchers have advocated for a prudent approach to the future integration of algorithmic explanations, recommending that such methods be regarded as supplementary decision-support tools pending the development of more robust interpretability methodologies [23].

### SHAP Features and Documentation Artifacts

To evaluate whether the model learned clinically meaningful signals or mere documentation artifacts, the top 10 SHAP features were manually mapped to the 14 guideline-defined SRMD indications (Table 1). Features such as “抗凝” (anticoagulation) and “外伤” (trauma) showed direct correspondence with guideline criteria (items 2 and 6, respectively). However, several high-ranking features raised concerns about confounding by documentation patterns. For example, “半月板 损伤” (meniscus injury) is a typically minor orthopedic condition, but its presence among the top 10 features suggests that the model relied on surface-level keyword co-occurrence rather than clinical severity stratification. Similarly, “抗感染” (anti-infection therapy) reflected the documentation of antibiotic use rather than the presence of sepsis (item 12), and “术后” (postoperative) appeared in notes following minor procedures that did not meet the >3-hour surgical threshold (item 7). These findings indicate that while the model captures genuine clinical concepts, some features act as confounding surrogates, highlighting the need for prospective validation against adjudicated clinical phenotypes and the integration of structured data (eg, procedure duration and laboratory values) to disambiguate proxy terms from true risk factors.

### Limitations

This study has several limitations. First, the dataset was limited in scale with a low proportion of positive samples; model training and validation were based solely on single-center data for a single condition (prophylactic PPI use for SRMD). Although the patient-wise 7:1.5:1.5 split resulted in a small independent test set (~10 patients), this partitioning strategy was deliberately chosen to prevent patient-level data leakage, which is methodologically preferable to record-level random splitting despite the resulting statistical volatility. The observed generalization gaps (ranging from 0.151 to 0.246 across models) reflect genuine performance degradation on unseen patients rather than inflated estimates from leaked records. Notably, the optimal model, CatBoost (balanced), exhibited relatively moderate gaps of 0.151, suggesting that model selection based on validation performance remains informative even under constrained sample sizes.

CIs for the cross-validated estimates were derived from a patient-level cluster bootstrap of out-of-fold predictions rather than from the single held-out test set, and the relatively wide intervals reflect the limited cohort size; the independent test set was never used in cross-validation or hyperparameter selection, so the held-out results reported in Table 4 are unaffected by any tuning procedure.

Second, a certain degree of covariate shift existed between the training/validation sets and the independent test set. Preliminary analysis suggested that this discrepancy may primarily stem from systematic differences in progress note length across different patients or admission stages, as well as interdepartmental variations in documentation style. While this partially reflects the unpredictable nature of data distribution in real-world environments for potential future integration, the present study did not systematically quantify the sources of shift or their impact on the consistency of model ranking performance due to sample size constraints. This observation indicates that future research should conduct cross-domain validation in multicenter, large-sample datasets and explore domain-adaptive fine-tuning strategies or distributionally robust optimization methods such as invariant risk minimization to enhance generalization stability under diverse clinical data distributions. Additionally, because of the limited test set sample size, CIs were derived from a patient-level cluster bootstrap of out-of-fold predictions rather than from the single held-out test set; future studies should evaluate the stability of estimates in larger samples. Third, the current classification rules relied on manual annotation and professional interpretation based on drug package inserts and clinical practice guidelines, a process characterized by low efficiency and inherent subjectivity; subsequent research may explore semiautomatic annotation or active learning strategies based on large language models to reduce labor costs. Finally, this study did not conduct prospective clinical validation; the time efficiency of the model in actual prescription indication screening workflows, pharmacist adoption rates, and the net benefit in terms of interception rates for inappropriate prescriptions remain to be evaluated through real-world studies. Given the retrospective, single-center design and absence of external validation, the proposed model should be regarded as a research prototype rather than a clinically deployable system.

### Conclusions

This study constructed and systematically evaluated a machine learning–based Chinese medical text classification framework for the automated identification of prophylactic PPI medication indications for SRMD from unstructured progress notes. The principal findings were as follows: first, in a severely class-imbalanced scenario with a positive rate of only 11.0%, the CatBoost model optimized with a class weight balancing strategy achieved a precision of 0.693, a recall of 0.765, and an AP of 0.751 for positive class identification. Among all compared models, it attained the highest note-level *F*_1_-score and the smallest generalization gap (0.151), although its AP was slightly lower than that of TextCNN (AP=0.797) and its precision was higher than that of BERT (precision=0.557); these results indicate that systematically tuned traditional machine learning models can effectively identify positive samples and provide a lightweight, interpretable alternative for future integration scenarios with limited computational resources or high requirements for explainability. Second, different models exhibited significant differences in precision-recall tendencies for positive class identification: CatBoost (with balanced strategy), characterized by high precision (0.693), is suitable for automated screening scenarios where low false positives are prioritized, whereas RandomForest (with class_weight=1:5 strategy), achieving the highest recall (0.809), is suitable for assisted screening scenarios. This suggests that model selection should match the false-positive vs false-negative cost structure of the specific clinical workflow. Third, SHAP-based interpretability analysis revealed key clinical features driving positive class identification, providing transparent and verifiable decision-making evidence for pharmacist review, which aligns with the regulatory requirements for decision transparency in health care settings. These findings provide an empirical basis for integrating NLP technologies into hospital prescription indication screening workflows and configuring model strategies according to specific scenarios. Future research could further explore the cross-disease generalization capability of large language models as zero-shot classifiers and conduct prospective clinical validation to evaluate the net benefit and pharmacist acceptance rate of the model in real-world prescription review workflows.

With the broad application prospects of machine learning in pharmaceutical practice, the responsibilities of pharmacists have expanded beyond traditional dispensing duties to encompass increasingly diverse domains: their scope of practice now encompasses not only the refined implementation of medication management and optimization of therapeutic regimens, but also the enhancement of patient medication safety and therapeutic benefit through specialized professional services [24].

In the future, AI-driven intelligent document processing will achieve deep integration of machine learning, NLP, optical character recognition, and robotic process automation technologies to construct efficient document workflow optimization solutions. This technical framework can automatically accomplish core tasks, including document classification, key information extraction, and physical document digitization. Combined with existing mature prescription review systems, it will substantially enhance the efficiency and accuracy of the full automation workflow for prescription indication screening through maximal reduction of manual intervention [25].

## Supplementary material

10.2196/103192 Multimedia Appendix 1 Comprehensive model performance.

## Abbreviations

AP: average precision
AUC: area under the curve
EHR: electronic health record
EMR: electronic medical record
NLP: natural language processing
PPI: proton pump inhibitor
PR-AUC: precision-recall area under the curve
ROC-AUC: receiver operating characteristic area under the curve
SHAP: Shapley additive explanations
SRMD: stress-related mucosal disease
TF-IDF: term frequency–inverse document frequency
TPE: tree‑structured Parzen estimator
TreeSHAP: tree-based Shapley additive explanations
